# Effect of continuous infusion of dexmedetomidine on blood loss in orthognathic surgery: a retrospective study

**DOI:** 10.1186/s40001-021-00551-5

**Published:** 2021-07-20

**Authors:** Chenyu Jin, Xiang Lv, Yu Sun, Hong Jiang

**Affiliations:** 1 Department of Anaesthesiology, Shanghai Ninth People’s Hospital, Shanghai JiaoTong University School of Medicine, Shanghai, China; 2 Present Address: Department of Anaesthesiology, Shanghai Ninth People’s Hospital, 639 Zhizaoju Road, Shanghai, China

**Keywords:** Dexmedetomidine, Blood loss, Orthognathic surgery

## Abstract

**Background:**

Patients with maxillofacial deformities require orthognathic surgeries to correct occlusion. The surgical procedure may lead to massive bleeding, which is associated with haematoma, respiratory obstruction, and asphyxia. Dexmedetomidine has been used in controlled hypotension and may reduce blood loss in orthognathic surgery. We conducted a retrospective cohort study to evaluate the effect of dexmedetomidine on blood loss in orthognathic surgeries.

**Methods:**

The primary outcome examined was blood loss, and secondary outcomes were postoperative haemoglobin level; intraoperative heart rate and blood pressure (T1: at incision; T2: 30 min after incision; T3: 60 min after incision; T4: 120 min after incision); dosage of fentanyl, remifentanil, urapidil, and esmolol; operation time; and incidence of allogeneic blood transfusion.

**Results:**

A total of 1247 patients were included in this study, and 540 patient pairs were matched via propensity score matching. There were significant decreases in mean blood loss, heart rate at T1–T4, blood pressure at T1, and remifentanil and esmolol dosage in the dexmedetomidine group compared with those in the control group. There was also a significant increase in the postoperative haemoglobin level of the dexmedetomidine group.

**Conclusions:**

Continuous infusion of dexmedetomidine can decrease blood loss in orthognathic surgery.

*Trial registration*: ChiCTR1800018794 (retrospectively registered)

Name of registry: Chinese Clinical Trial Registry

Date of registration: 2018/10/09

URL: www.chictr.org.cn/showproj.aspx?proj=30612

## Background

Patients with maxillofacial deformities require orthognathic surgeries (among others, Le Fort I osteotomy and bilateral sagittal split ramus osteotomy) to correct occlusions or their physical appearance [1, 2]. The rich blood supply and deep surgical site of the oral and maxillofacial region often lead to increased bleeding and a limited visual field during osteotomy of the maxilla, thus increasing the risk posed by surgery. Intraoperative haemorrhage can be associated with many postoperative complications, such as respiratory obstruction, and even asphyxia [3]. Controlled hypotension has been previously used to decrease bleeding in many maxillofacial surgeries [4, 5]. Many anaesthetic and vasoactive drugs have been used successfully to achieve controlled hypotension [6]. Dexmedetomidine is a potent, highly selective α_2_-adrenoceptor agonist that may provide anti-sympathetic analgesia and sedation without respiratory depression [7], and it has been successfully used for controlled hypotension in other surgical procedures [8].

The sedative and analgesia-sparing effects of dexmedetomidine are associated with its effects on the central nervous system in the locus coeruleus and spinal cord dorsal horn neurons [9]. Dexmedetomidine is an α_2_-adrenoreceptor agonist. It primarily inhibits norepinephrine release and causes attenuation of excitation in the central nervous system [10]. Binding of postsynaptic receptors by α_2_-agonists leads to inhibition of sympathetic activity, which decreases the blood pressure (BP) and heart rate (HR) and results in sedation [11].

This retrospective cohort study aimed to determine the efficacy of dexmedetomidine for managing intraoperative blood loss, perioperative haemodynamics, anaesthetic drug requirements, the incidence of blood transfusion, and length of hospital stay in orthognathic surgeries.

## Methods

### Study design

The study is registered at Chinese Clinical Trial Registry (ChiCTR1800018794). Ethical approval (SH9H-2019-T244-2) was obtained from the Shanghai Ninth People’s Hospital Research Ethics Committee. We conducted a retrospective cohort study on patients who underwent orthognathic surgeries between March 2017 and August 2018. Institutional review board approval was obtained before the initiation of this study. Patients with a complete medical history and surgical records were included if they were over 18 years of age and underwent elective orthognathic surgery. Patients were excluded from the analysis if they had a history of any of the following: allergies to intraoperative-related drugs, heart-related diseases (New York Heart Association class III or higher), severe pulmonary disease (asthma, chronic obstructive pulmonary disease), or severe liver and kidney dysfunction. Each controlled hypotension strategy is different, and there is currently no unified standard strategy for controlled hypotension. Indications for dexmedetomidine include tracheal intubation and sedation during mechanical ventilation for patients undergoing general anaesthesia, and the administration of dexmedetomidine depends on the judgement of senior anaesthesiologists. Patients treated with continuous dexmedetomidine were allocated to the dexmedetomidine group, while patients treated without continuous dexmedetomidine were allocated to the control group.

### Anaesthesia procedures and perioperative medication

Upon arrival in the operating room, HR, BP, and oxygen saturation were monitored by electrocardiography, non-invasive BP monitoring, and pulse oximetry, respectively, before an intravenous cannula was inserted. Induction of anaesthesia was similar in every patient, comprising the intravenous administration of 2 mg midazolam, 2–4 µg/kg fentanyl, 1.5–2.5 mg propofol, and 0.15–0.2 mg/kg cisatracurium. Patients underwent endotracheal intubation, invasive BP monitoring, and deep-vein catheterisation during anaesthesia induction. According to the requirements of the surgeon, most patients underwent acute normovolemic hemodilution (ANH) after anaesthesia and before the beginning of the main steps of surgery [12]. Patients in the dexmedetomidine group were administered a 0.5–1 µg/kg loading dose of dexmedetomidine, and 200–400 mL autologous blood was rapidly extracted before incision. Crystalloid fluids were simultaneously infused into patients to supplement the circulating blood volume, dilute the blood, and reduce the loss of visible blood components during surgery. The extracted autologous blood was transfused back to patients before the end of the operation. This has been reported to be an efficacious, safe, and protective method to reduce the concentration of circulating erythrocytes with minimal effects on clotting factors and platelets [13, 14].

At the beginning of the operation, controlled hypotension was initiated, and vital signs were closely monitored. Each patient received 4–8 mg/kg/h propofol and 6–18 µg/kg/h remifentanil intravenously and 2–3% sevoflurane via inhalation. To ensure intraoperative sedation and prevent intraoperative awareness, the bispectral index (BIS) of all patients was controlled at the same level of 10–30. All patients were maintained under deep anaesthesia. Patients were administered 0.1 mg fentanyl before incision and later again, if necessary. Patients in the dexmedetomidine group received continuous dexmedetomidine at 0.2–0.5 µg/kg/h. Narcotic drugs and analgesics, including dexmedetomidine, were initially infused 10 min before incision. Mean arterial BP was controlled between 50–60 mmHg. If mean arterial BP was too high, 5 mg urapidil or 5–10 mg esmolol was used. If mean arterial BP was < 50 mmHg, 6 mg ephedrine was used. If a patient was bradycardic during the operation, the infusion of dexmedetomidine was discontinued, and the patient was excluded from the study. Patients were administered 0.5 g tranexamic acid after incision. Intraoperative fluid management was individualised based on the actual condition of the patients, and it was affected by physiological requirements, fasting supplementation, intraoperative loss, and other factors. Rehydration rate changed from fast to slow, and 15 − 20 ml/kg crystal or colloid fluids were administered intravenously in the first hour. During the operation, colloid fluids or blood products would be administered after communicating with a superior anaesthesiologist and surgeon based on the actual condition of the patients. Allogeneic blood transfusion was performed if necessary, at the discretion of the surgeon and anaesthesiologist. Several clinical measures were used to indicate allogeneic blood transfusion, and these included haemoglobin level < 70 g/L, haematocrit < 30%, and blood loss > 15% of estimated blood volume [15]. In addition, the anaesthesiologist and surgeon communicated and arrived at a consensus before allogeneic transfusion was performed. Administration of all narcotic drugs and analgesics, including dexmedetomidine, was discontinued 15 to 30 min before the end of the operation. When the operation was close to completion, the patients’ BP would be increased to the normal level.

The volume of blood loss was measured by the circulating nurses and recorded in medical history data by anaesthesiologists. The method of measuring blood loss was applied as follows: the total volume of liquid in the suction canister, plus the added weight of wet medical gauze and cloth surgical linen, minus the volume of saline used for irrigation during the procedure.

### Data collection

Study data were obtained from intraoperative records and the electronic medical record. The primary outcome was intraoperative blood loss. The secondary outcomes were postoperative haemoglobin level (patients who received allogeneic blood transfusions were excluded); intraoperative HR and BP (both measured at four points: T1, at incision; T2, 30 min after incision; T3, 60 min after incision; T4, 120 min after incision); dosage of fentanyl, remifentanil, urapidil, and esmolol; operation time; and incidence of allogeneic blood transfusion.

A standard operating procedure (SOP) was established to train relevant researchers, research assistants, and statistical analysts. The researchers completed case report forms according to the SOP. Two researchers independently entered the data, and the research assistant checked and generated data query forms (DQFs). If the research data were lost in the electronic database, the researchers went to the medical history room to retrieve the paper medical history data for verification. Problems in DQFs would be modified and resolved after discussion. Most of the problems could be solved using information from the electronic database or paper medical history records. Some problems (such as missing key data) were considered to be at risk of causing bias after discussion, and we would exclude the patients associated with these problems. Once the modification was complete, data was locked and passed to the statistical analyst.

### Data analysis

Propensity score matching (PSM) was introduced to reduce bias due to confounding factors, which might affect surgical decision-making in patients with maxillofacial deformities. Confounding factors included age, height, weight, sex, preoperative haemoglobin level, prothrombin time, activated partial thromboplastin time, ANH, preoperative HR and BP, BIS, and volume of crystalloid and colloid fluids. Patients who underwent orthognathic surgeries were matched at a 1:1 ratio with a calliper width equal to 0.02, resulting in the same number of patients in both groups. The t-test was used for parametric scale variables. Scale variables were tested for normality with the Kolmogorov–Smirnov test. The Chi-square exact test was used for nominal variables. Statistical analyses were performed using SPSS version 25 for Windows (IBM, Armonk, NY). Differences between the two groups were expressed as difference in means, standard deviation (SD), mean difference (MD), or odds ratio (OR) with 95% confidence intervals (CI). Statistical significance was defined as a p-value < 0.05.

## Results

**Fig. 1:**
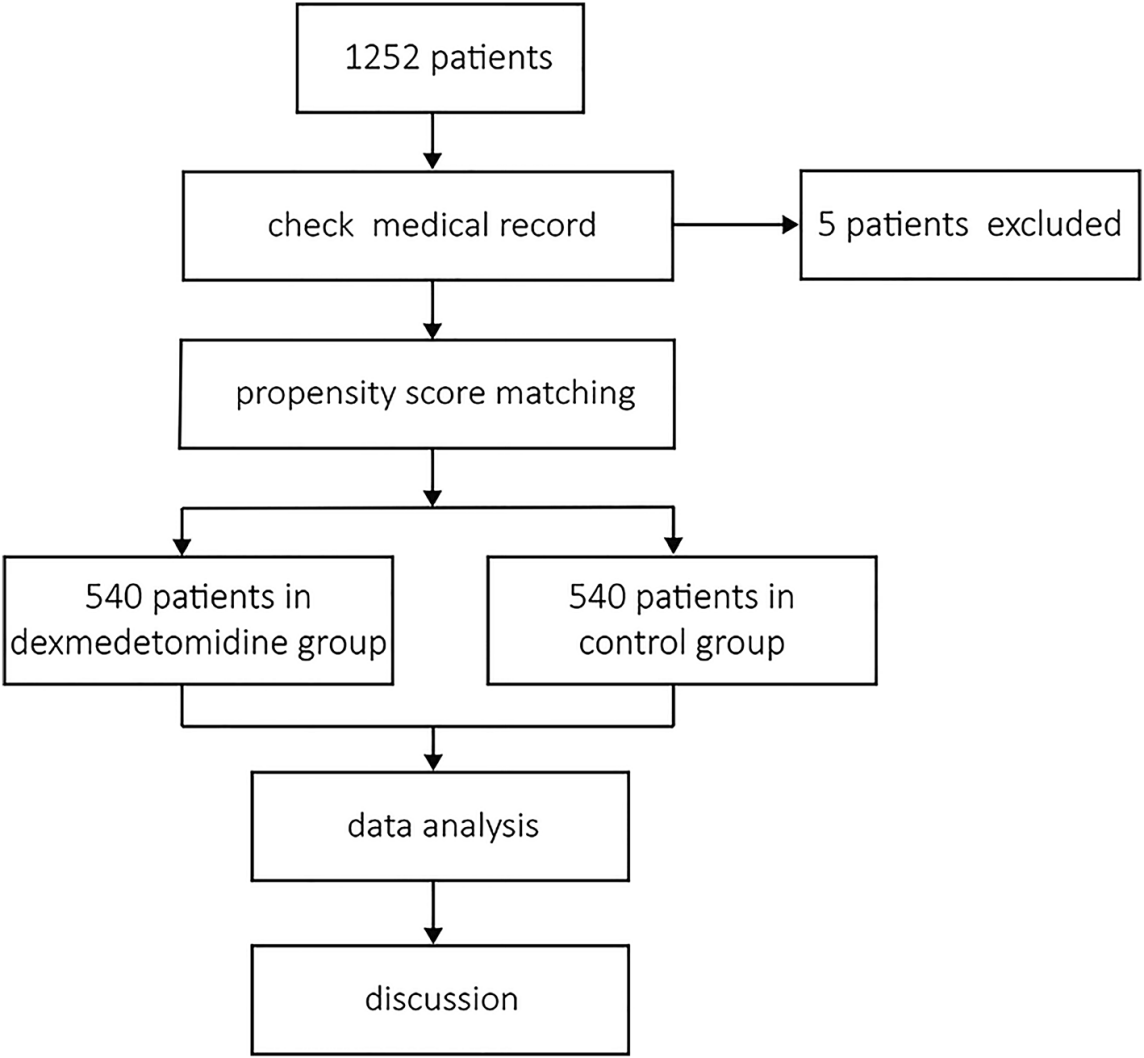
Flow diagram of the study design

Initially, 1252 patients were identified for analysis in the defined study time period based on the inclusion criteria. After the researchers checked the medical records, five patients were excluded because infusions of dexmedetomidine were discontinued during operations. A total of 1,247 patients were finally included for analysis. All patients were classified as American Society of Anaesthesiologists class I–II. Dexmedetomidine was used continuously in 560 patients, and they were allocated to the dexmedetomidine group. Dexmedetomidine was administered at a maintenance dose of 0.2–0.5 µg/kg/h. Dexmedetomidine was never used in 687 patients, and they were allocated to the control group. PSM was conducted to randomise and control variables, and 540 pairs of patients who underwent orthognathic surgeries were matched. No statistically significant differences were noted at baseline between the PSM-adjusted groups. The study process is shown in Fig. 1. Baseline patient characteristics are shown in Table 1.

**Table 1:** Baseline patient characteristics after PSM

Variable	Dexmedetomidine group	Control group	P Value
Number	540	540	/
Age (y), mean ± SD	23.66 ± 4.37	23.55 ± 4.27	0.694
Height (cm), mean ± SD	166.30 ± 8.52	166.49 ± 8.31	0.699
Weight (kg), mean ± SD	59.20 ± 11.09	59.20 ± 11.58	0.993
Gender (M/F)	188/352	190/350	0.899
Preoperative haemoglobin (g/L), mean ± SD	128.92 ± 14.67	128.82 ± 14.91	0.915
PLT (× 10^9^/L), mean ± SD	240.25 ± 47.93	241.29 ± 54.16	0.739
PT (s), mean ± SD	11.39 ± 0.93	11.37 ± 0.81	0.660
APTT (s), mean ± SD	28.36 ± 2.38	28.17 ± 2.39	0.174
ANH(mL), mean ± SD	353.51 ± 128.23	352.22 ± 139.49	0.874
Patients with ANH/patients without ANH	496/44	490/50	0.517
Preoperative MAP (mmHg)	86.47 ± 8.40	86.68 ± 8.55	0.681
Preoperative HR (bpm)	80.80 ± 9.41	81.03 ± 10.04	0.711
BIS	17.70 ± 4.99	17.64 ± 4.95	0.854
Crystalloid fluids (mL)	2780.46 ± 520.06	2804.81 ± 555.07	0.457
colloid fluids (mL)	388.89 ± 350.81	387.96 ± 372.95	0.966

*PSM* Propensity Score Matching, *SD* standard deviation, *BMI* body mass index, *PLT* platelet, *PT* prothrombin time, *APTT* activated partial thromboplastin time, *ANH* acute normovolemic hemodilution, *MAP* mean arterial pressure, *bpm* beat per minute, *BIS* bispectral index

There was a significant decrease in mean blood loss, HR at T1–T4, BP at T1, dosages of remifentanil and esmolol in the dexmedetomidine group. There was a significant increase in postoperative haemoglobin level in the dexmedetomidine group. However, there was no significant difference in operation time, intraoperative BP at T2–T4, incidence of allogeneic blood transfusion, or dose of fentanyl or urapidil. The results of the primary and secondary outcomes are shown in Table 2.

**Table 2:** Primary and secondary outcomes after PSM

Variable	Dexmedetomidine group	Control group	95% CI	P Value
Blood loss (mL), mean ± SD	670.83 ± 273.23	732.31 ± 286.88	− 94.93 to − 28.03	< 0.001
Postoperative haemoglobin (g/L), mean ± SD	103.92 ± 14.60	100.23 ± 16.18	1.72 to 5.66	< 0.001
Intraoperative HR (bpm), mean ± SD				
T1	70.89 ± 11.05	78.06 ± 13.10	− 8.62 to − 5.73	< 0.001
T2	72.93 ± 11.28	79.72 ± 11.58	− 8.15 to − 5.42	< 0.001
T3	71.87 ± 10.38	79.66 ± 11.29	− 9.09 to − 6.50	< 0.001
T4	69.79 ± 10.54	78.40 ± 10.67	− 9.88 to − 7.34	< 0.001
Intraoperative BP (mmHg), mean ± SD				
T1	60.22 ± 9.99	62.50 ± 10.48	− 3.50 to − 1.06	< 0.001
T2	55.94 ± 7.69	56.74 ± 8.89	− 1.80 to 0.18	0.112
T3	54.74 ± 5.89	54.90 ± 7.05	− 0.94 to 0.60	0.673
T4	56.19 ± 7.92	57.02 ± 8.92	− 1.84 to 0.18	0.109
Operation time (h), mean ± SD	3.57 ± 1.12	3.62 ± 1.22	− 0.19 to 0.09	0.499
Fentanyl (mg), mean ± SD	0.35 ± 0.07	0.35 ± 0.07	− 0.01 to 0.01	0.881
Remifentanil (mg), mean ± SD	1.23 ± 0.45	1.49 ± 0.61	− 0.32 to − 0.19	< 0.001
Incidence of allogeneic blood transfusion (transfused /not transfused)	32/508	35/505	/	0.801
Urapidil (mg), mean ± SD	12.60 ± 5.88	12.41 ± 5.48	− 0.48 to 0.87	0.574
Esmolol (mg), mean ± SD	18.04 ± 10.02	25.37 ± 10.88	− 8.58 to − 6.08	< 0.001

*PSM* Propensity Score Matching, *MD* mean difference, *OR* odds ratio, *CI* confidence interval, *SD* standard deviation, *ICU* Intensive Care Unit

T1: at incision; T2: 30 min after incision; T3: 60 min after incision; T4: 120 min after incision

## Discussion

The results showed a significant decrease in the mean total calculated blood loss in the dexmedetomidine group compared to that in the control group. This may have been due to the haemodynamic effect of dexmedetomidine. Continuous infusion of dexmedetomidine led to decreased HR and BP, which was caused by a negative feedback loop of norepinephrine [16]. Recent studies have also suggested that dexmedetomidine can decrease blood loss throughout several different surgical procedures [8, 17]. In previous studies, the surgical field of vision, which significantly affects blood loss, was considered to be directly related to decreased HR [18, 19]. There is evidence that decreasing mean arterial pressure below 70 mmHg increases intraoperative bleeding due to local vasodilation [20], but decreased HR is strongly correlated with cardiac output, which is associated with the operative field of vision [19]. In contrast to α_2_ agonists, inhalational anaesthetics lead to vasodilatory effects [21] and reflex tachycardia. Furthermore, opioids are less effective than dexmedetomidine in reducing HR. Decreased mean arterial pressure without controlled HR does not lead to improved visibility or lessened bleeding [22, 23]. In addition, the vasoconstrictive effect of intravenous dexmedetomidine has been demonstrated in animal models [24, 25]. Furthermore, studies have provided evidence that intravenous dexmedetomidine has similar vasoconstrictive effects on human arteries and veins [26]. Contraction of the peripheral vessels caused by intravenous dexmedetomidine would further promote surgical site visualisation and reduction of bleeding. Improved surgical field of vision has been mentioned in other studies [23, 27]. This finding is important since it is closely related to blood loss and ease of operation for surgeons. Unfortunately, because this is a retrospective study, the intraoperative visual field could not be assessed using a numerical rating scale or other quantitative methods. Therefore, we could not verify whether the intraoperative field of vision in orthognathic surgery was improved as in other surgeries. Reduced bleeding will lead to fewer complications, such as haematoma, respiratory obstruction, and asphyxia, and will further promote the patient's postoperative recovery.

There was a significant increase in postoperative haemoglobin level in patients treated with intraoperative dexmedetomidine. Patients who underwent allogeneic blood transfusions during operations were excluded because the level of postoperative haemoglobin would be affected. Reduced intraoperative blood loss increased levels of postoperative haemoglobin and improved postoperative safety. Postoperative anaemia is associated with dizziness, tinnitus, fatigue, hypoxia, and other side effects [28]. Postoperative acute anaemia is correlated with an increased risk of injury to major organs, such as the brain, heart, and kidney [29], and elevated haemoglobin levels will help avoid these risks.

There was a significant decrease in the intraoperative HR of patients treated with intraoperative dexmedetomidine. The current study also revealed that deliberate hypotension with lower HR could reduce intraoperative blood loss and improve the surgical field [30, 31]. Dexmedetomidine decreases HR [32], specifically causing a 16–30% decrease from baseline at plasma drug concentrations > 1–3 ng/mL [33, 34]. There was a significant decrease in intraoperative BP at T1 in patients treated with intraoperative dexmedetomidine. The decreased BP may be caused by transiently elevated plasma concentration, and it might further contribute to the reduction of intraoperative blood loss in the early surgical procedure. There was no significant difference in the average arterial pressure at most time points due to human control. The anaesthesiologist maintained the target BP level after the mean BP reached the target point. In addition, the requirement for esmolol was decreased because of the effect of reducing HR.

There was no difference in operation time between the considered groups. Although the amount of blood loss was reduced by dexmedetomidine, the operation time was not shortened. Similar studies have also shown that dexmedetomidine improves the quality of the surgical field without significantly affecting operation time [8, 35]. No difference in the requirement of fentanyl was observed between the groups, but the remifentanil requirement in the dexmedetomidine group was significantly decreased. Studies have shown that long-term and high-dose use of opioids may produce some side effects, such as hyperalgesia, nausea and vomiting, and emergence agitation [36, 37]. The reduced dosage of remifentanil may help alleviate any hyperalgesia and reduce postoperative nausea and vomiting.

There was no significant difference in the incidence of allogeneic blood transfusion between the groups. This might be due to the low transfusion incidence of this surgical procedure. Most patients who undergo orthognathic surgeries do not need blood transfusion, with the exception of those who experience massive bleeding. Studies have also reported that ANH significantly reduces allogeneic blood transfusion [12, 38].

In addition, the dose of crystalloid fluids seemed to be large for an average 3.5-h duration of surgery. This may have been caused by heavy bleeding and ANH. Patients were required to be transfused with a large amount of crystalloid fluids after a predetermined amount of autologous blood was rapidly withdrawn to supplement blood volume and maintain stable vital signs [39]. This protective measure increased crystalloid fluid dosage. ANH is a routine protective measure in orthognathic surgery in our hospital, and most of the patients were treated with this technique.

Although the results are promising, there are some limitations to this study. First, the retrospective nature of this study has inherent limitations and potential interference factors regarding data integrity and homogeneity. However, we have strictly followed the criteria for inclusion and exclusion and have used a rigorous statistical approach to avoid bias. Second, this study was a single-centre retrospective study, which may have led to selection bias. We expanded the sample size to minimise bias. Third, assessment of visualisation was pointed out; however, it could not be assessed because of the retrospective nature of this study. Surgical site visualisation requires the surgeon’s evaluation using any method, such as the numerical rating scale or other methods, but we cannot obtain such data from the electronic database or paper medical history. We plan to conduct further studies to elaborate on this aspect. Fourth, different drugs, including propofol, remifentanil, and sevoflurane, were administered, and this may have affected the accuracy of our conclusions. We used various methods to ensure the reliability of the conclusions, such as expanding the sample size, PSM, and strict data management. Fifth, ANH is a protective measure of autologous transfusion, leading to blood loss and blood dilution. It may cause bias and affect the results. As a routine in orthognathic surgery, it is almost impossible to use this as an exclusion criterion. However, the rate of ANH between the two groups was balanced, and PSM included ANH as the confounding factor to reduce bias. Finally, different surgeons use different approaches; thus, the methodology of each operation is different. For example, some surgeons might think that preoperative ANH is necessary, while others might not.

The present study showed that dexmedetomidine decreases blood loss in orthognathic surgeries, and we plan to conduct a randomised controlled study in the future.

## Conclusion

Continuous infusion of dexmedetomidine decreases blood loss in orthognathic surgeries. Dexmedetomidine also increases postoperative haemoglobin and decreases intraoperative HR.

## Data Availability

The datasets used and/or analysed during the current study are available from the corresponding author on reasonable request.

## Abbreviations

HR: Heart rate
BP: Blood pressure
BIS: Bispectral index
ANH: Acute normovolemic hemodilution
SOP: Standard operating procedure
DQF: Data query form
PSM: Propensity score matching
SD: Standard deviation
MD: Mean difference
OR: Odds ratio
CI: Confidence interval
